# Does Mind-Wandering Explain ADHD-Related Impairment in Adolescents?

**DOI:** 10.1007/s10578-023-01557-2

**Published:** 2023-06-29

**Authors:** Tycho J. Dekkers, Ajda Flisar, Adrian Karami Motaghi, Alexandra Karl, Matilda A. Frick, Bianca E. Boyer

**Affiliations:** 1 Department of Psychology, University of Amsterdam, Amsterdam, the Netherlands; 2 Department of Child and Adolescent Psychiatry, University of Groningen, University Medical Center Groningen, Groningen, the Netherlands; 3 Accare Child Study Center, Groningen, the Netherlands; 4 Levvel, Academic Center for Child and Adolescent Psychiatry, Amsterdam, the Netherlands; 5 Department of Child and Adolescent Psychiatry, Amsterdam University Medical Center (AUMC), Amsterdam, the Netherlands; 6 Department of Psychology, Stockholm University, Stockholm, Sweden; 7 Department of Medical Sciences, Uppsala University, Uppsala, Sweden; 8 Psychologenpraktijk Kuin, Haarlem, the Netherlands; 9 Department of Developmental and Educational Psychology, Leiden University, Leiden, the Netherlands

**Keywords:** Mind-wandering, Attention-deficit, Hyperactivity disorder (ADHD), Adolescence, Impairment

## Abstract

Currently, diagnostic criteria for ADHD mainly reflect behavioral symptoms, neglecting internal phenomena like mind-wandering. Recent studies found that mind-wandering explains impairment beyond ADHD symptoms in adults. To better capture ADHD-related impairment in adolescents, we aimed to elucidate whether mind-wandering is associated with impairments that are prevalent in adolescents (i.e., risk-taking behavior, homework problems, emotional dysregulation, and general impairment) beyond ADHD symptoms. Furthermore, we sought to validate the Dutch translation of the Mind Excessively Wandering Scale (MEWS). We assessed a community sample of 626 adolescents on ADHD symptoms, mind-wandering, and the impairment domains. The Dutch MEWS had good psychometric properties. Mind-wandering was linked to general impairment and emotional dysregulation beyond ADHD symptoms, but was not linked to risk-taking behavior and homework problems beyond ADHD symptoms. Internal psychological phenomena such as mind-wandering may add to the behavioral symptoms of ADHD in explaining part of the impairment that adolescents with ADHD characteristics experience.

**Supplementary Information:**

The online version contains supplementary material available at 10.1007/s10578-023-01557-2.

## Introduction

Attention-deficit/hyperactivity disorder (ADHD) is characterized by developmentally inappropriate levels of inattention and/or hyperactivity/impulsivity. It is very common in childhood, affecting about 5–7% of children and adolescents [59]. ADHD characteristics manifest across different settings and interfere with social, academic, and later occupational functioning [2, 48]. Relative to childhood, the symptoms of ADHD are less visible from the outside in adolescence: hyperactivity and impulsivity typically diminish and problems with inattention and sluggish cognitive tempo are often causing most impairment [4, 24]. ADHD may therefore be underreported in adolescence as youth with primarily inattentive symptoms are less likely to be recognized [13, 46] and less likely to receive evidence-based treatment [61].

Currently, diagnostic criteria for ADHD reflect behavioral symptoms and do not address psychological phenomena that are experienced more internally, such as day-dreaming or mind-wandering [2, 7]. The reliance on behavioral symptoms to classify ADHD may result in a construct that does not sufficiently explain the accompanied impairments. In this study, mind-wandering was considered as an internal mechanism that may explain impairment (i.e., risk-taking behavior, homework problems, emotional dysregulation, and general impairment) beyond the behavioral symptoms of ADHD.

Mind-wandering is defined as a shift in attention from a currently attended task to internal thoughts [53] and is often proposed as an internal psychological process explaining inattention. Although related, mind-wandering slightly differs from the traditional DSM-5 symptoms of inattention in that it can be defined as “internal distractibility” and is more covert, whereas the inattention symptoms in the DSM-5 merely reflect distractibility to external stimuli, which could be considered more overt behavior [5, 45]. Mind-wandering is ubiquitous in daily life: deliberate or spontaneous drifting away from a task to internal task-unrelated thoughts occurs in almost half of people’s waking hours [37, 57]. A growing body of research has drawn a positive relationship between mind-wandering and ADHD, in particular the inattentive presentation of ADHD [5, 36]. A recent study in children similarly observed that children with ADHD were more susceptible to mind-wandering than their unaffected peers [44]. In adults with ADHD, spontaneous mind-wandering, relative to deliberate mind-wandering, was specifically elevated relative to adults without ADHD and was positively related to ADHD severity [53]. Excessive spontaneous mind-wandering and ADHD symptoms share neurobiological correlates in the default mode network, and both were related to executive-functioning problems [9]. Hence, excessive spontaneous mind-wandering may underpin ADHD symptomatology and in turn contribute to impairment, making it a highly relevant construct of investigation.

Thus far, not many studies have examined mind-wandering in youth. A first study in college students used an experience sampling technique: Students with and without a childhood history of ADHD were asked to report on- and off-task thoughts during a simple attention task. The frequency of task-unrelated thoughts was higher in adolescents with a childhood history of ADHD relative to controls [54]. More recently, a positive correlation was found between self-reported mind-wandering and self-reported symptoms of inattention and hyperactivity/impulsivity (but *not* parent-reported ADHD symptoms) in adolescents with ADHD [25]. Based on this small number of studies, a positive association between mind-wandering and ADHD symptoms in adolescents with ADHD is likely, comparable to what has been observed in adults.

A stronger emphasis on internal processes, such as mind-wandering, may help to explain functional impairment of individuals with ADHD. For example, in adults with ADHD, mind-wandering explained functional impairment across major life domains and well-being beyond ADHD symptoms [45, 46]. In children with ADHD, mind-wandering contributed to deficits in working memory and emotion regulation beyond the effects of ADHD symptoms [26]. Moreover, a recent review including eleven studies, mostly on adults, also reported a positive correlation between mind-wandering and functional impairment in ADHD [41]. These include decreased performances at school or work. Here, we test the hypothesis that the subjective experience of mind-wandering may provide a better prediction of ADHD-associated impairments in adolescents than the traditional ADHD inattention and hyperactive/impulsive symptoms [9].

In this study, we focused on general impairment and on three specific domains in which adolescents with ADHD typically experience substantial impairment and which are theoretically related to mind-wandering: risk-taking behavior, homework problems, and emotional dysregulation. First, ADHD is consistently associated with increased engagement in risk-taking behaviors during adolescence, such as risky driving, risky sexual behavior, substance abuse, criminal behavior, and financial risk taking [17, 50]. Moreover, in young adults, mind-wandering added to the explanation of risk-taking behavior beyond ADHD symptoms [46].

Second, ADHD symptoms are associated with decrements in homework performance and adolescents with ADHD more often have significant problems completing and managing their homework relative to controls [14, 40, 58]. As increased mind-wandering during lectures is negatively related to short- and long-term academic performance and decreased performance on cognitive tasks, the hypothesized contribution of mind-wandering to homework problems is plausible [51, 60].

Third, emotional dysregulation concerns the inability to modify the behavioral, physiological, and experiential correlates of emotions and is characterized by both increased emotionality and a decreased ability to regulate emotions [11, 28]. Emotional dysregulation is common in adolescents with ADHD [19, 43] and may serve as a major contributor to impairment in social life [10]. Moreover, excessive spontaneous mind-wandering is positively related to symptoms of anxiety and depression in adolescents [22], and with emotional liability in young adults [45], suggesting a link between mind-wandering and emotional dysregulation [5]. In sum, excessive mind-wandering is linked to several behaviors that often cause impairment in adolescents with ADHD. As such, mind-wandering may serve as an independent contributor to impairment beyond the behavioral manifestations of ADHD.

In the present pre-registered study, we aimed to investigate whether mind-wandering explains ADHD-related impairment above and beyond ADHD symptoms in a large sample of typically developing adolescents (*N* = 626). We used the recently developed Mind Excessively Wandering Scale (MEWS), which is a self-report rating scale to assess excessive spontaneous mind-wandering. The MEWS has potential utility as a screening tool in clinical practice to assist diagnostic assessment [45, 46], and displays good psychometric properties in children and adults [26, 45]. As a preliminary aim, we first focused on assessing psychometric properties of our Dutch translation of the MEWS. Second, we investigated whether mind-wandering was linked to functional impairments often experienced by adolescents with ADHD (general impairment, risk-taking behavior, homework problems, and emotional dysregulation) above and beyond ADHD symptoms. In line with the research discussed above, we expected independent contributions of mind-wandering to all domains of impairment.

## Methods

### Preregistration

This study was preregistered on AsPredicted (#15669: https://aspredicted.org/bj73g.pdf). Note that this pre-registration also contains information about a previously published study [16].

### Participants

Participants were 626 adolescents ages 16–22 (see Table 1) from secondary schools in the Netherlands. The majority attended a so-called “multimedia college”, a school with an educational focus on creativity, which increased the likelihood of including adolescents on the high end of the ADHD symptomatology continuum [30, 33]. This was confirmed by the relatively high prevalence rates of (self-reported) lifetime ADHD diagnoses. A minimum age of 16 years was the only inclusion criterion, and there were no exclusion criteria. The intelligence of all participants was likely to be average or above as participating schools were of an average educational level or higher.

**Table 1:** Sample characteristics (mean scores and standard deviations)

	Total sample	Sample 1	Sample 2	Re-test sample
*n*	626	312	315	202
Age (16–22)	17.18 (1.22)	17.24 (1.30)	17.11 (1.13)	17.07 (1.15)
Gender (% M/F/T/NA)	35.6/63.3/0.2/1.0	33.7/65.1/0/1.3	37.8/61.3/0.3/0.6	32.2/67.3/0/0.5
No lifetime self-reported psychiatric disorder	385 (61.5%)	195 (62.5%)	191 (60.6%)	139 (68.8%)
Lifetime ADHD diagnosis	105 (16.8%)	49 (15.7%)	56 (17.8%)	28 (13.9%)
ADHD symptoms (0–50.5)	15.28 (9.11)	15.18 (8.79)	15.40 (9.43)	15.07 (8.94)
Mind Wandering (0–36)	14.28 (8.27)	14.10 (8.07)	14.46 (8.47)	13.82 (7.75)/14.53 (8.09)*
Impairment (0–35)	11.26 (8.10)	11.24 (8.23)	11.28 (7.98)	10.98 (7.80)
Risk-Taking Behavior (0–73)		18.90 (12.62)		19.40 (10.66)
Homework Problems (0–56)			16.67 (10.60)	14.75 (9.96)
Emotionality (10–46)		30.93 (7.29)		31.00 (6.90)
Emotion regulation (20–99)		68.18 (11.64)		68.32 (11.00)

*M* Male, *F* Female, *T* Transgender, *NA* not available

*First value in this cell represents mean (and standard deviation) of the re-test sample at the first administration, the second value represents the mean (and standard deviation) at re-test

Minimum and maximum values are depicted in parentheses

To not burden participants, not all questionnaires were administered to all participants: 312 adolescents (i.e., sample 1) completed questionnaires on ADHD symptoms, general impairment, mind-wandering, emotional dysregulation, and risk-taking behavior and 315 adolescents (i.e., sample 2) completed questionnaires on ADHD symptoms, general impairment, mind-wandering, and homework problems^1^. Further, 202 adolescents from both subsamples participated in a follow-up on the MEWS to measure test–retest reliability. Adolescents reported on previous (lifetime) diagnoses of mental disorders, with 105 (16.8%) indicating a previous diagnosis of ADHD (any presentation). Furthermore, 61.5% reported never having received a diagnosis of a mental disorder, 8.3% reported a generalized anxiety disorder, 3.2% reported a diagnosis of autism spectrum disorder, 11.2% reported having been diagnosed with depression, 2.9% with an eating disorder, 1.8% with obsessive–compulsive disorder, 0.2% with a tic disorder, and 1.0% with a substance use disorder. Primarily, the ethnic identity of the adolescents was Dutch (92.5% Dutch, 7.3% Surinamese, 4.3% Antillean, 4.6% Moroccan, 4.8% Turkish, 2.7% Kurdish, 19.2% other).^2^

### Measures

#### Mind Excessively Wandering Scale (MEWS)

Mind-wandering was assessed using a Dutch translation of the MEWS [45]. The MEWS was translated into Dutch by two native Dutch speakers who were also proficient in English, and then translated back into English by two native English speakers also proficient in Dutch. The Dutch items can be found in the Supplementary Materials, original English items were taken from Mowlem et al. [46]. During both steps, disagreements were solved by discussion between the translators, also including two of the authors (TJD, BEB). The scale consists of 12 items, which cover various aspects of mind-wandering and are based on experience of adolescents’ reports on mind-wandering. Items were assessed on a 4-point Likert scale ranging from “not at all or rarely” to “nearly all of the time or constantly”. An example of an item is: “I find it hard to switch my thoughts off”. Scores could range between 0 and 36, with higher scores indicating more mind-wandering. In an earlier study, the MEWS showed good internal consistency (α = 0.93) and was able to differentiate between adults with ADHD and controls [46].

#### ADHD self-Report Scale

ADHD symptoms over the last six months were measured using the Dutch ADHD self-report scale for adults (ASRS) [38]. The questionnaire consists of 23 items that are assessed on a 4-point Likert scale ranging from “never or rarely” to “very often”. An example of an item is “I get bored easily”. A minimum of 0 and maximum score of 69 could be obtained, with higher scores indicating more ADHD symptoms. Items reflect symptoms of ADHD, based on the Diagnostic and Statistical Manual of Mental Disorders (DSM-IV-TR) [1]. Each symptom was evaluated with one item, except for five symptoms with double items, which were averaged for the analyses. The ASRS has good internal and external validity for individuals between 18 and 70 years old [39]. In our sample, internal consistency was high with *α* = 0.91.

#### Impairment Rating Scale

The Dutch translation of the Impairment Rating Scale (IRS) [18] was originally administered to parents or teachers, but was for the sake of the current study reformulated into a self-report questionnaire consisting of 6 items. Items were assessed on a visual analogue scale and covered various aspects of impairment: peer relations, parent relationship, academic performance, self-esteem, effects on family functioning, and overall impairment. The visual analogue scale ranged from 0 (“no problem”) to 6 (“extreme problem”). An example of an item is “to what extent are your problems affecting your relationships with other children”. Total scores on this measure range from 0 to 36, where higher scores reflect more impairment. The psychometric properties of this measure are good with an average test–retest reliability ranging from 0.76 to 0.90 [18]. The current study proved good internal consistency of this scale with *α* = 0.88.

#### Risk-Taking Behavior Self-Report Questionnaire

To measure risk-taking behavior, the self-reported risk-taking behavior questionnaire was administered [16, 62]. This questionnaire was based on three existing questionnaires: the risk taking behavior questionnaire [49], the adolescent version of the Domain-Specific Risk-Taking scale (DOSPERT) [6] and the Adolescent Risk Taking Inventory (AdoRTI) [27]. This scale consists of 28 items, which were assessed on a 5-point Likert scale. Participants indicated the frequency of risky behavior on a range from “never” to “every week”. An example of an item is “How often do you use drugs?”. A minimum score of 0 and maximum score of 112 could be obtained, where higher scores reflected more risk-taking behavior. In the current study, this questionnaire showed good internal consistency with *α* = 0.86.

#### Homework Problems Checklist (HPC)

The Dutch translation of the HPC was used to assess the extent of various problems regarding school homework of adolescents [3]. This questionnaire, originally created to be answered by parents, was adjusted to a self-report format. It consists of 20 items that were answered on a 4-point Likert scale. Items such as “I easily get frustrated by homework assignments” were answered on a range from “never” to “very often”. The total score of the checklist ranged from 0 to 60, and higher scores indicated more homework problems. The original version of this measure has good psychometric properties with a Cronbach’s α ranging from 0.76 to 0.92 [3, 23]. In our sample, the HPC showed high internal consistency (*α* = 0.92).

#### Emotions Questionnaire

The Dutch translation of the Emotions Questionnaire [52] was applied to assess emotional dysregulation, operationalized as emotionality and emotion regulation. The questionnaire consists of 30 items regarding five emotions (anger, fear, sadness, worry, and exuberance) measured on a 4-point Likert scale, with scores ranging from 0 to 90. Ten items (two per emotion) concern emotionality, such as “I experience strong feelings when I get angry” and 20 items (four per emotion) concern emotion regulation, such as “If I am angry and my teacher tells me to calm down, I can control myself”. Higher scores indicate higher emotionality and better emotion regulation, respectively. Adolescents answered both scales on a 4-point Likert scale ranging from “Does not apply to me at all” to “Applies very well to me”. Moderate internal consistency was found for this questionnaire (*α* = 0.62) [52]. In our sample, internal consistency was good for the emotionality subscale (α = 0.82) as well as for the emotion regulation subscale (*α* = 0.89).

### Procedure

Prior to the assessment, participants were provided with information about the study in class. After providing written informed consent, participants filled out the questionnaires on a laptop in their classroom. While the participants completed the questionnaires, two research assistants and the teacher answered emerging questions. Adolescents without a laptop filled out the questionnaires with paper-and-pencil. The assessment lasted for approximately 30 min. The study was approved by the IRB of the University of Amsterdam.

### Data Analysis

The analyses were performed using SPSS version 27. Outliers were detected by the Median Absolute Deviation (MAD) method [42], using a moderately conservative MAD of 2.5. The median is more robust to outliers than the traditional approach of deviation from the mean. As pre-registered, we report analyses with and without outliers. When less than 25% of items of a questionnaire was missing, the total score was imputed by the mean score of the filled-in items of that participant on that questionnaire. When more than 25% was missing, data on that questionnaire of that participant were discarded. Cronbach’s α was calculated to investigate the internal consistency of the MEWS. Correlations for the psychometric properties of the MEWS were calculated with total scores on the MEWS and ASRS. Normality was checked using the Shapiro–Wilk test [21]. Hierarchical (blockwise entry) regression models for confirmatory analyses were bootstrapped to 2000 samples and all values were standardized: ADHD symptoms were entered in the first step of the regression and mind-wandering in the second step. All analyses were pre-registered except for the analyses regarding emotional dysregulation, which are therefore considered exploratory. An a priori power analysis was performed using G*power [20]. To obtain a medium effect size, with alpha level of 0.05 and power of 0.80 our study would require 68 participants. As our sample size was larger than required the current study had sufficient power.

## Results

### Assumptions

All scores on the questionnaires violated the assumption of normality (all Shapiro Wilk’s > 0.92, *p*s < 0.004). An inspection of the scatterplots for scores on all questionnaires showed a linear relationship with ADHD and MEWS scores. According to the residual scatterplots, the assumptions of homoscedasticity and linearity were met for scores on all questionnaires. Multicollinearity was not violated, as all variance inflation factors (VIF) were below 2.52 [21].

### Outliers

Following the MAD method, no outliers were detected for mind-wandering at first and second administration. Eighteen outliers were detected for ADHD symptoms, 7 for risk-taking behavior, 11 for homework problems, 4 for emotionality, 14 for emotion regulation, and 5 for general impairment. As pre-registered, analyses were executed with and without outliers. Only one of the results changed in terms of significance when outliers were removed, this was indicated explicitly. Here, we report analyses including outliers; all main analyses excluding outliers can be found in the supplementary materials.

### Descriptive Information

Correlations between all variables can be found in Table 2. In addition, we found that those adolescents with a self-reported lifetime diagnosis of ADHD scored higher on overall ADHD symptoms (*p* < 0.001, *95%BCI* [− 18.00, − 15.35]), inattention (*p* < 0.001, *95%BCI* [− 4.62, − 3.90]), hyperactivity/impulsivity (*p* < 0.001, *95%BCI* [− 3.65, − 2.84]) and mind-wandering (*p* < 0.001, *95%BCI* [− 13.04, − 10.30]) than those without a lifetime ADHD diagnosis.

**Table 2:** Correlations between the study variables

	1	2	3	4	5	6	7	8	9
1.Mind-wandering	1	0.77***	0.74***	0.66***	0.57***	0.30***	0.60***	0.53***	− 0.31***
2.ADHD Symptoms		1	0.93***	0.89***	0.55***	0.46***	0.79***	0.48***	− 0.25***
3.Inattention			1	0.66***	0.54***	0.43***	0.81***	0.44***	− 0.23***
4.Hyperactivity/impulsivity				1	0.47***	0.40***	0.62***	0.41***	− 0.23***
5.Impairment					1	0.26***	0.55***	0.60***	− 0.33***
6.Risk-taking behavior						1	N/A	0.15**	− 0.18**
7.Homework problems							1	N/A	N/A
8.Emotionality								1	− 0.24***
9.Emotion regulation									1

All correlations reflect Spearman’s rho coefficients based on standardized values; N/A = not available

*p < 0.05, **p < 0.01, ***p < 0.001

### Psychometric Evaluation of the MEWS

At baseline, the MEWS shows high internal consistency for the total sample (Cronbach’s α = 0.93). This did not improve by omitting items. Each item appears to fit well with the total score according to item-total correlations (correlations > 0.63; see Table S3 in Supplementary Materials for all item-total correlations). Furthermore split-half reliability was high (*r*_*sb*_ = 0.90). Examining convergent validity at baseline for the total sample, the MEWS correlated moderately to highly with total ADHD scores (*r*_*s*_ = 0.77, *p* < 0.001), inattention (*r*_*s*_ = 0.69, *p* < 0.001) and hyperactivity (*r*_*s*_ = 0.60, *p* < 0.001). There was an interval of 2–3 months between baseline and follow-up measurement on the MEWS, test–retest reliability was moderate/high (*r*_*s*_ = 0.77, *p* < 0.001).

### Hierarchical Regression Analyses

For all models, *F*-values, *p*-values, *R*^*2*^, change in *R*^*2*^, bootstrapped *B* coefficients, and bootstrapped confidence intervals are reported in Table 3. Table 4 includes results from the analyses when inattention was used as predictor instead of overall ADHD symptoms, to test whether this affected the results. As we found differences between boys and girls on risk-taking behavior (higher in boys), mind wandering (higher in girls), emotionality (higher in girls) and general impairment (higher in girls), we also conducted our analyses for boys and girls separately (see Supplementary Materials 4 for detailed results). When results differed between boys and girls, we also indicated this in the section below. Age was unrelated to all study variables (*r*s = -0.06 to 0.12) and thus left out of all further analyses.

**Table 3:** Hierarchical regression analyses

		Outcome *bootstrapped B*	95% BCI	*F (df)*	*R*^2^	△*R*^2^
		General impairment (*n* = 626)				
Step 1	ADHD	0.57***	[0.51, 0.63]	294.35*** (1,623)	0.32	
Step 2	ADHD	0.27***	[0.17, 0.36]	190.88*** (2,622)	0.38	0.06***
	MEWS	0.39***	[0.28, 0.49]			
		Risk-taking behaviors (*n* = 312)				
Step 1	ADHD	0.47***	[0.35, 0.59]	78.59*** (1,310)	0.20	
Step 2	ADHD	0.53***	[0.34, 0.72]	39.84*** (2,309)	0.21	0.003
	MEWS	− 0.08	[− 0.26, 0.10]			
		Homework problems (*n* = 315)				
Step 1	ADHD	0.77***	[0.69, 0.84]	527.54*** (1,313)	0.63	
Step 2	ADHD	0.75***	[0.63, 0.87]	263.06*** (2,312)	0.63	0.000
	MEWS	0.02	[− 0.10, 0.15]			
		Emotionality (*n* = 312)				
Step 1	ADHD	0.48***	[0.36, 0.60]	85.52*** (1,310)	0.22	
Step 2	ADHD	0.16*	[− 0.01, 0.31]	63.34*** (2,309)	0.29	0.08***
	MEWS	0.43***	[0.30, 0.56]			
		Emotion regulation (*n* = 312)				
Step 1	ADHD	− 0.24***	[− 0.37, − 0.11]	17.88*** (1,310)	0.06	
Step 2	ADHD	− 0.03	[− 0.20, 0.15]	14.56*** (2,309)	0.09	0.03**
	MEWS	− 0.28**	[− 0.45, − 0.11]			

*Note*. All variables reflect sum scores. The table shows standardized bootstrapped regression coefficients where general impairment, risk-taking behaviors, homework problems, emotionality, and emotion regulation are regressed on the ADHD symptom score and the MEWS score

Step 1 of each regression model shows association of ADHD symptom score with all outcome measures.

Step 2 includes two predictors, ADHD and MEWS, and shows the additional contribution of mind-wandering in association with all five outcome measures.

BCI = Bootstrapped Confidence Interval, Df = degrees of freedom, MEWS = Mind Excessively Wandering Scale.

*p < 0.05, **p < 0.01, ***p < 0.001.

**Table 4:** Hierarchical regression analyses (inattention instead of ADHD)

		Outcome *Bootstrapped B*	95% BCI	*F (df)*	*R*^2^	△*R*^2^
		General impairment (*n* = 626)				
Step 1	Inattention	0.55***	[0.49, 0.61]	266.83*** (1,623)	0.30	
Step 2	Inattention	0.24***	[0.14, 0.33]	187.64*** (2,622)	0.38	0.08***
	MEWS	0.42***	[0.32, 0.52]			
		Risk-taking behaviors (*n* = 312)				
Step 1	Inattention	0.40***	[0.29, 0.52]	58.73*** (1,310)	0.16	
Step 2	Inattention	0.39***	[0.21, 0.56]	29.30*** (2,309)	0.16	0.00
	MEWS	0.02	[− 0.17, 19]			
		Homework problems (*n* = 315)				
Step 1	Inattention	0.82***	[0.75, 0.89]	653.76*** (1,313)	0.68	
Step 2	Inattention	0.80***	[0.69, 0.89]	326.80*** (2,312)	0.68	0.00
	MEWS	0.04	[− 0.07, 0.15]			
		Emotionality (*n* = 312)				
Step 1	Inattention	0.43***	[0.32, 0.55]	69.34*** (1,310)	0.18	
Step 2	Inattention	0.07	[− 0.07, 0.22]	61.14*** (2,309)	0.28	0.10***
	MEWS	0.49***	[0.35, 0.62]			
		Emotion regulation (*n* = 312)				
Step 1	Inattention	− 0.23***	[− 0.36, − 0.11]	17.73*** (1,310)	0.05	
Step 2	Inattention	− 0.03	[− 0.19, 0.13]	14.58*** (2,309)	0.09	0.03**
	MEWS	− 0.28**	[− 0.44, − 0.11]			

All variables reflect sum scores

The table shows standardized bootstrapped regression coefficients where general impairment, risk-taking behaviors, homework problems, emotionality, and emotion regulation are regressed on the inattention symptom score and the MEWS score

Step 1 of each regression model shows the association of the inattention symptom score with all outcome measures

Step 2 includes two predictors, inattention and MEWS, and shows the additional contribution of mind-wandering in association with all five outcome measures

*BCI* bootstrapped confidence interval, *Df* degrees of freedom, *MEWS* mind excessively wandering scale

*p < 0.05, **p < 0.01, ***p < 0.001

### General Impairment

When mind-wandering was added to the model with only ADHD symptoms, this resulted in a significant increase in explained variance; the model containing ADHD-symptoms and mind-wandering explained 38% of the variance. This was an increase of 6% as compared to the explained variance of the first model containing ADHD-symptoms only. After mind-wandering was added to the model, there still was a smaller but significant association between ADHD symptoms and general impairment, and also a significant association between mind-wandering and general impairment was found. Results were similar when boys and girls were analyzed separately (see Supplementary Materials 4).

### Risk-Taking Behavior

Adding mind-wandering to the first model, with only ADHD symptoms, did not result change the explained variance. We did find that the first model (with ADHD symptoms only) was significantly associated with risk-taking behavior, explaining 20% of the variance. Adding mind-wandering to the model did not change the association between ADHD symptoms and risk-taking behavior, and there was no association between mind-wandering and risk-taking behavior. Results were similar when boys and girls were analyzed separately (see Supplementary Materials 4).

### Homework Problems

When mind-wandering was added to the first model with only ADHD symptoms, the explained variance did not change. The first model with ADHD symptoms only, was significantly associated with homework problems, explaining 63% of the variance. When mind-wandering was added to the model, the relation between ADHD symptoms and homework problems did not change, and there was no association between mind-wandering and homework problems. Results were similar when boys and girls were analyzed separately (see Supplementary Materials 4).

### Emotionality

Adding mind-wandering to the model with only ADHD symptoms resulted in a significant increase in explained variance; the second model explained 29% of the variance. In the first step of the analyses, we found that ADHD symptoms were significantly associated with emotionality, explaining 22% of the variance. When mind-wandering was added to the model, we found a smaller but still significant association between ADHD symptoms and emotionality (note that this association was no longer significant when outliers were excluded, see supplementary materials), and then also found a significant association between mind-wandering and emotionality. For the gender-specific analyses, the link between ADHD symptoms and emotionality was no longer significant for boys (see Supplementary Materials 4).

### Emotion Regulation

Adding mind-wandering to the model with only ADHD symptoms, resulted in a significant increase in explained variance; the model explained 9% of the variance. In the first step of the analyses, we found that ADHD symptoms were significantly associated with emotion regulation (i.e., higher levels of ADHD symptoms were related to lower scores on emotion regulation), explaining 6% of the variance. In the second step, with mind-wandering added to the model, we no longer found a significant association between ADHD symptoms and emotion regulation. Instead, we found a significant association between mind-wandering and emotion regulation (i.e., higher levels of mind-wandering were related to poorer emotion regulation). For boys, there was no significant association of either ADHD and mind-wandering with emotion regulation, whereas the results for girls were similar as the overall results reported above (see Supplementary Materials 4).

### Inattention as Predictor Instead of ADHD

As mind-wandering seems more closely related to inattention than to hyperactivity/impulsivity, we repeated all analyses with inattention as predictor instead of the overall ADHD symptom score. Findings were highly similar in terms of statistical significance. The only result that differed was for emotionality: when mind-wandering was added to the model, inattention was no longer significantly associated with emotionality (see Table 4).

## Discussion

Mind-wandering concerns a shift in attention from a currently attended task to internal thoughts. The main goal of this study was to elucidate whether mind-wandering adds to the symptoms of ADHD as described by the DSM-5 in explaining general impairment and domain-specific functional impairments (i.e., risk-taking behavior, homework problems, emotionality, and emotion regulation) that are highly prevalent in adolescents with ADHD. To obtain a broad range of symptoms, we examined these associations in a population sample of adolescents, with oversampling of adolescents with above average number of ADHD symptoms as they were recruited from a school with a focus on creativity. To provide an adequate measurement of mind-wandering, we first investigated the psychometric properties of our Dutch translation of the Mind Excessively Wandering Scale (MEWS). Internal consistency and split-half reliability were excellent, and item-total correlations were all moderate. Convergent validity with ADHD, inattention, and hyperactivity subscales was moderate to high, and test–retest reliability was moderate to high.

Using this psychometrically sound version of the MEWS in a large sample of Dutch adolescents, we showed that, even though the effect sizes were rather small, mind-wandering explained variance above and beyond overall ADHD characteristics in general impairment, emotionality, and emotion regulation. However, mind-wandering did not add to ADHD symptoms in explaining risk-taking behavior and homework problems. Crucially, findings were highly similar when the added value of mind-wandering was tested in models with inattention only (instead of overall ADHD symptoms), and most associations were very similar for boys and girls. These mixed results suggest that mind-wandering indeed adds to ADHD symptoms in explaining some co-occurring impairments (mainly related to emotion dysregulation) in adolescents, but provide only partial support for our hypothesis that mind-wandering might be a better predictor of impairments linked to ADHD than the core behavioral symptoms.

Despite rather small effect sizes, which were potentially only statistically significant because of our large sample, the finding that excessive mind-wandering in adolescents is associated with emotionality and emotional dysregulation above and beyond the inattention symptoms of ADHD aligns with previous findings in children with ADHD [26]. Relatedly, the link between ADHD and emotional processing was aggravated by mind-wandering in adults [31]. Several adaptive emotion-regulation strategies, such as cognitive reappraisal and situation modification, require conscious and voluntary control of thoughts [29]. Mind-wandering may hamper these top-down processes and lead to elevated emotionality and deficient emotion regulation. Further, the link between emotional dysregulation and mind-wandering could be related to the frustration associated with spontaneous mind-wandering and to the content of mind-wandering. Although bidirectional links between mind-wandering and emotional dysregulation are likely, a hallmark study suggests that mind-wandering, especially when the mind wanders about neutral or unhappy topics, causes unhappiness [37]. Uncontrollable and quickly shifting thoughts often tend to be ruminative and painted by negative content such as fears and failures [32, 47, 55]. A promising avenue for future research would therefore be to investigate whether mind-wandering is relevant for understanding the well-established link between ADHD and internalizing disorders [35].

Mind-wandering was expected to predict difficulties during academic tasks. However, mind-wandering did not add to ADHD symptoms in predicting academic problems (in the present study operationalized as homework problems). This is in line with earlier findings that showed that mind-wandering did not add to the explained variance of ADHD symptoms on academic achievement [26]. This is somewhat surprising given that ADHD and mind-wandering are related to increased baseline levels of activation in the Default Mode Network and reduced deactivation of this network during cognitive tasks [9]. The finding further implies that the well-established link between ADHD and homework problems [14] could be sufficiently explained by the behavioral symptoms of ADHD. Alternatively, Frick and colleagues [26] suggest that the link between mind-wandering and academic problems may exist but could be mediated by deficits in working memory. To disentangle these interpretations, future studies on ADHD and mind-wandering could include measures of neurocognitive functions.

Mind-wandering was not independently associated with risk-taking behavior, which contrasts earlier work in adults [46]. Risk-taking behavior in individuals with ADHD is most often linked to impulsivity and hyperactivity [15, 50], which are the behavioral symptoms that are most distinct from mind-wandering. However, some manifestations of risk-taking behavior (e.g., risky driving) may be particularly related to mind-wandering. The difference between our and previous findings could be explained by the difference in sample. Potentially, only very excessive forms of mind-wandering are associated to risk-taking behavior, which may be more frequent in the clinical ADHD sample included by Mowlem and colleagues [46] than in our community sample.

The current study has several strengths, such as the large sample, the rigorous investigation of the psychometric properties of our main measure (the MEWS), the measurement of both general and specific indicators or ADHD-related impairment and the preregistration of our methods and design. Notwithstanding these strengths, several limitations warrant consideration. First, we emphasize that our data are cross-sectional and that we are therefore unable to make any causal inferences. Second, we only relied on self-report measures that potentially lead to socially desirable or otherwise biased responses [56]. Recent research shows promise of more objective measures of mind-wandering, such as EEG or eye-tracking [8, 12, 34]. Third, we did not obtain multi-informant reports, meaning that biases in self-reporting may have played a role. However, the expected strong correlation between ADHD symptoms and mind-wandering that we observed provides us with confidence about the validity of our measurement.

This study shows that considering internal psychological phenomena such as mind-wandering may add to the behavioral symptoms of ADHD in explaining at least some of the impairment that adolescents with ADHD characteristics experience. Our study, along with other recent work, further demonstrates that adolescents are perfectly capable of reporting their own mind-wandering. Considering such internal processes may do more justice to the experience and impairment of individuals with ADHD than when only their externally observable behavior is considered. Although highly important, we conclude that research in this area is still in its infancy, and future studies are highly needed to elucidate to what extent consideration of mind-wandering may improve assessment, prevention and treatment of adolescents with ADHD.

## Summary

To better capture ADHD-related impairment in adolescents, we aimed to elucidate whether mind-wandering was associated with impairments that are prevalent in adolescents (i.e., risk-taking behavior, homework problems, emotional dysregulation, and general impairment) beyond ADHD symptoms. Mind-wandering was linked to general impairment and emotional dysregulation beyond ADHD symptoms, but was not linked to risk-taking behavior and homework problems beyond ADHD symptoms. Internal psychological phenomena such as mind-wandering may add to the behavioral symptoms of ADHD in explaining part of the impairment that adolescents with ADHD characteristics experience.

## Supplementary Information

Below is the link to the electronic supplementary material.

Supplementary file1 (DOCX 45 KB)

## Data Availability

Anonymized data and materials are available upon request via the corresponding author.

## Footnotes

1 One participant accidentally completed both batteries, which is why the participant number adds up to 627 instead of 628 participants.

2 Participants could give multiple answers to which socio-cultural group they identified with, therefore the sum exceeds 100%.

## References

[1] American Psychiatric Association (2000) Diagnostic and statistical manual of mental disorders, 4th revised edition (DSM-IV-TR). American Psychiatric Association, Washington DC

[2] American Psychiatric Association (2013) Diagnostic and Statistical Manual of Mental Disorders, 5th edn. American Psychiatric Association, Washington DC

[3] Anesko K, Schoiock G, Ramirez R, Levine F (1987) The homework problem checklist: assessing children’s homework difficulties. Behav Assess 9(2):179–185

[4] Becker SP, Langberg JM (2013) Sluggish cognitive tempo among young adolescents with ADHD: relations to mental health, academic, and social functioning. J Atten Disord 17(8):681–689. 10.1177/1087054711435411 22441891 10.1177/1087054711435411

[5] Biederman J, Lanier J, DiSalvo M, Noyes E, Fried R, Woodworth KY, Faraone SV (2019) Clinical correlates of mind wandering in adults with ADHD. J Psychiatric Res 117:15–23. 10.1016/j.jpsychires.2019.06.012 10.1016/j.jpsychires.2019.06.012 31272014

[6] Blais A-R, Weber EU (2006) A domain-specific risk-taking (DOSPERT) scale for adult populations. Judgm Decis Mak 1(1):33–47

[7] Bozhilova N, Michelini G, Jones C, Kuntsi J, Rubia K, Asherson P (2021) Context Regulation of Mind Wandering in ADHD. J Atten Disord 25(14):2014–2027. 10.1177/1087054720956714 32942934 10.1177/1087054720956714 PMC8527550

[8] Bozhilova N, Cooper R, Kuntsi J, Asherson P, Michelini G (2020) Electrophysiological correlates of spontaneous mind wandering in attention-deficit/hyperactivity disorder. Behav Brain Res. 10.1016/j.bbr.2020.112632 32361038 10.1016/j.bbr.2020.112632 PMC7303944

[9] Bozhilova N, Michelini G, Kuntsi J, Asherson P (2018) Mind wandering perspective on ADHD. Neurosci Biobehav Rev 92:464–476. 10.1016/j.neubiorev.2018.07.010 30036553 10.1016/j.neubiorev.2018.07.010 PMC6525148

[10] Bunford N, Evans SW, Wymbs F (2015) ADHD and emotion dysregulation among children and adolescents. Clin Child Fam Psychol Rev 18(3):185–217. 10.1007/s10567-015-0187-5 26243645 10.1007/s10567-015-0187-5

[11] Cole PM, Martin SE, Dennis TA (2004) Emotion regulation as a scientific construct: methodological challenges and directions for child development research. Child Dev 75(2):317–333. 10.1111/J.1467-8624.2004.00673.X 15056186 10.1111/j.1467-8624.2004.00673.x

[12] Conrad C, Newman A (2021) Measuring mind wandering during online lectures assessed with EEG. Front Human Neurosci. 10.3389/fnhum.2021.697532 10.3389/fnhum.2021.697532 PMC8381606 34434097

[13] Cuffe SP, Moore CG, McKeown RE (2005) Prevalence and correlates of ADHD symptoms in the national health interview survey. J Atten Disord 9(2):392–401. 10.1177/1087054705280413 16371662 10.1177/1087054705280413

[14] De Vries M, Van der Oord S, Evans SW, DuPaul GJ, Boyer BE (2022) The homework problems checklist: psychometric properties and usefulness in teens with and without ADHD. School Mental Health. 10.1007/s12310-022-09548-9

[15] Dekkers TJ, de Water E, Scheres A (2022) Impulsive and risky decision-making in adolescents with attention-deficit/hyperactivity disorder (ADHD): the need for a developmental perspective. Curr Opin Psychol 44:330–336. 10.1016/j.copsyc.2021.11.002 34953445 10.1016/j.copsyc.2021.11.002

[16] Dekkers TJ, Huizenga HM, Bult J, Popma A, Boyer BE (2021) The importance of parental knowledge in the association between ADHD symptomatology and related domains of impairment. Eur Child Adolesc Psychiatry 30(4):657–669. 10.1007/s00787-020-01579-4 32699991 10.1007/s00787-020-01579-4 PMC8041705

[17] Dekkers TJ, Popma A, Agelink van Rentergem JA, Bexkens A, Huizenga HM (2016) Risky decision making in attention-deficit/hyperactivity disorder: a meta-regression analysis. Clin Psychol Rev 45:1–16. 10.1016/j.cpr.2016.03.001 26978323 10.1016/j.cpr.2016.03.001

[18] Fabiano GA, Pelham WE, Waschbusch DA, Gnagy EM, Lahey BB, Chronis AM, Burrows-MacLean L (2006) A practical measure of impairment: Psychometric properties of the impairment rating scale in samples of children with attention deficit hyperactivity disorder and two school-based samples. J Clin Child Adolesc Psychol 35(3):369–385. 10.1207/s15374424jccp3503_3 16836475 10.1207/s15374424jccp3503_3

[19] Faraone SV, Rostain AL, Blader J, Busch B, Childress AC, Connor DF, Newcorn JH (2019) Practitioner Review: Emotional dysregulation in attention-deficit/hyperactivity disorder—implications for clinical recognition and intervention. J Child Psychol Psychiatry 60(2):133–150. 10.1111/jcpp.12899 29624671 10.1111/jcpp.12899

[20] Faul F, Erdfelder E, Lang A, Buchner A (2007) G*Power 3: a flexible statistical power analysis program for the social, behavioral, and biomedical sciences. Behav Res Methods 39:175–191 17695343 10.3758/bf03193146

[21] Field AP (2013) Discovering statistics using IBM SPSS Statistics, 4th edn. Sage, Los Angeles

[22] Figueiredo T, Lima G, Erthal P, Martins R, Corção P, Leonel M, Mattos P (2020) Mind-wandering, depression, anxiety and ADHD: Disentangling the relationship. Psychiatry Res. 10.1016/j.psychres.2020.112798 31991281 10.1016/j.psychres.2020.112798

[23] Foley RM, Epstein M (2005) Evluation of the homework problem checklist with students with behavior disorders. Special Serv Schools 7(1):79–90. 10.1300/j008v07n01_05

[24] Franke B, Michelini G, Asherson P, Banaschewski T, Bilbow A, Buitelaar JK, Reif A (2018) Live fast, die young? A review on the developmental trajectories of ADHD across the lifespan. Eur Neuropsychopharmacol 28:1059–1088. 10.1016/j.euroneuro.2018.08.001 30195575 10.1016/j.euroneuro.2018.08.001 PMC6379245

[25] Fredrick JW, Becker SP (2021) Sluggish cognitive tempo symptoms, but not ADHD or internalizing symptoms, are uniquely related to self-reported mind-wandering in adolescents with ADHD. J Atten Disord 25(11):1605–1611. 10.1177/1087054720923091 32463332 10.1177/1087054720923091 PMC8019063

[26] Frick MA, Asherson P, Brocki KC (2020) Mind-wandering in children with and without ADHD. Br J Clin Psychol 59(2):208–223. 10.1111/bjc.12241 31880828 10.1111/bjc.12241

[27] Goueta N, Gershy N, van Hoorn J, Pollak Y (2021) The relations between parental knowledge, ADHD Symptoms and risky adolescent behavior at two time points—ProQuest. Israelian J Psychiatry 58(2):62–70

[28] Gross JJ (1999) Emotion regulation: past, present, future. Cogn Emot 13(5):551–573. 10.1080/026999399379186

[29] Gross JJ (2015) Emotion regulation: current status and future prospects. Psychol Inq 26(1):1–26. 10.1080/1047840X.2014.940781

[30] Healey D, Rucklidge JJ (2006) An investigation into the relationship among ADHD symptomatology, creativity, and neuropsychological functioning in children. Child Neuropsychol 12(6):421–438. 10.1080/09297040600806086 16952888 10.1080/09297040600806086

[31] Helfer B, Boxhoorn S, Songa J, Steel C, Maltezos S, Asherson P (2021) Emotion recognition and mind wandering in adults with attention deficit hyperactivity disorder or autism spectrum disorder. J Psychiatr Res 134:89–96. 10.1016/j.jpsychires.2020.12.059 33373778 10.1016/j.jpsychires.2020.12.059

[32] Helfer B, Cooper RE, Bozhilova N, Maltezos S, Kuntsi J, Asherson P (2019) The effects of emotional lability, mind wandering and sleep quality on ADHD symptom severity in adults with ADHD. Eur Psychiatry 55:45–51. 10.1016/j.eurpsy.2018.09.006 30384113 10.1016/j.eurpsy.2018.09.006

[33] Hoogman M, Stolte M, Baas M, Kroesbergen E (2020) Creativity and ADHD: A review of behavioral studies, the effect of psychostimulants and neural underpinnings. Neurosci Biobehav Rev 119:66–85. 10.1016/J.NEUBIOREV.2020.09.029 33035524 10.1016/j.neubiorev.2020.09.029

[34] Hutt S, Krasich K, Mills C et al (2019) Automated gaze-based mind wandering detection during computerized learning in classrooms. User Model User-Adap Inter 29:821–867. 10.1007/s11257-019-09228-5

[35] Jensen PS, Hinshaw SP, Kraemer HC, Lenora N, Newcorn JH, Abikoff HB, Vitiello B (2001) ADHD comorbidity findings from the MTA study: comparing comorbid subgroups. J Am Academy Child Adolesc Psychiatry 40(2):147–158 10.1097/00004583-200102000-00009 11211363

[36] Jonkman LM, Markus CR, Franklin MS, Van Dalfsen JH (2017) Mind wandering during attention performance: Effects of ADHD-inattention symptomatology, negative mood, ruminative response style and working memory capacity. PLoS ONE 12(7):e0181213. 10.1371/journal.pone.0181213 28742115 10.1371/journal.pone.0181213 PMC5524389

[37] Killingsworth MA, Gilbert DT (2010) A wandering mind is an unhappy mind. Science 330(6006):932. 10.1126/SCIENCE.1192439/21071660 10.1126/science.1192439

[38] Kooij, J. J. S., & Buitelaar, J. K. (1997). Zelfrapportage Vragenlijst over aandachtsproblemen en hyperactiviteit [Self-report Questionnaire for attentional problems and hyperactivity].

[39] Kooij JJS, Buitelaar J, van den Oord E, Furer J, Rijnders T, Hodiamont P (2005) Internal and external validity of attention-deficit hyperactivity disorder in a population-based sample of adults. Psychol Med 35(6):817–827. 10.1017/S003329170400337X 15997602 10.1017/s003329170400337x

[40] Langberg JM, Arnold LE, Flowers AM, Altaye M, Epstein JN, Molina BSG (2010) Assessing homework problems in children with ADHD: validation of a parent-report measure and evaluation of homework performance patterns. Sch Ment Heal 2(1):3–12. 10.1007/s12310-009-9021-x 10.1007/s12310-009-9021-x PMC3085461 21544228

[41] Lanier J, Noyes E, Biederman J (2021) Mind wandering (Internal Distractibility) in ADHD: a literature review. J Atten Disord 25(6):885–890. 10.1177/1087054719865781 31364436 10.1177/1087054719865781

[42] Leys C, Ley C, Klein O, Bernard P, Licata L (2013) Detecting outliers: do not use standard deviation around the mean, use absolute deviation around the median. J Exp Soc Psychol 49(4):764–766. 10.1016/j.jesp.2013.03.013

[43] Martel MM (2009) Research review: a new perspective on attention-deficit/hyperactivity disorder: emotion dysregulation and trait models. J Child Psychol Psychiatry 50(9):1042–1051. 10.1111/j.1469-7610.2009.02105.x 19508495 10.1111/j.1469-7610.2009.02105.x

[44] Merrill BM, Raiker JS, Mattfeld AT et al (2022) Mind-wandering and childhood ADHD: experimental manipulations across laboratory and naturalistic settings. Res Child Adolesc Psychopathol 50:1139–1149. 10.1007/s10802-022-00912-6 35247108 10.1007/s10802-022-00912-6 PMC11103794

[45] Mowlem FD, Agnew-Blais J, Pingault JB, Asherson P (2019) Evaluating a scale of excessive mind wandering among males and females with and without attention-deficit/hyperactivity disorder from a population sample. Sci Rep 9(1):1–9. 10.1038/s41598-019-39227-w 30816143 10.1038/s41598-019-39227-w PMC6395591

[46] Mowlem FD, Skirrow C, Reid P, Maltezos S, Nijjar SK, Merwood A, Asherson P (2019) Validation of the mind excessively wandering scale and the relationship of mind wandering to impairment in adult ADHD. Journal of Attention Disorders 23(6):624–634. 10.1177/1087054716651927 27255536 10.1177/1087054716651927 PMC6429624

[47] Nayda DM, Takarangi MKT (2021) The cost of being absent: Is meta-awareness of mind-wandering related to depression symptom severity, rumination tendencies and trauma intrusions? J Affect Disord 292:131–138. 10.1016/j.jad.2021.05.053 34119868 10.1016/j.jad.2021.05.053

[48] Nigg JT (2013) Attention-deficit/hyperactivity disorder and adverse health outcomes. Clin Psychol Rev 33(2):215–228. 10.1016/J.CPR.2012.11.005 23298633 10.1016/j.cpr.2012.11.005 PMC4322430

[49] Pat-Horenczyk R, Peled O, Miron T, Brom D, Villa Y, Chemtob CM (2007) Risk-taking behaviors among Israeli adolescents exposed to recurrent terrorism: provoking danger under continuous threat? Am J Psychiatry 164(1):66–72. 10.1176/appi.ajp.164.1.66 17202546 10.1176/ajp.2007.164.1.66

[50] Pollak Y, Dekkers TJ, Shoham R, Huizenga HM (2019) Risk-taking behavior in attention deficit/hyperactivity disorder (ADHD): a review of potential underlying mechanisms and of interventions. Curr Psychiatry Rep 21(5):33. 10.1007/s11920-019-1019-y 30903380 10.1007/s11920-019-1019-y

[51] Randall JG, Oswald FL, Beier ME (2014) Mind-wandering, cognition, and performance: a theory-driven meta-analysis of attention regulation. Psychol Bull 140:1411–1431 25089941 10.1037/a0037428

[52] Rydell AM, Thorell LB, Bohlin G (2007) Emotion regulation in relation to social functioning: an investigation of child self-reports. Eur J Develop Psychol 4(3):293–313. 10.1080/17405620600783526

[53] Seli P, Carriere JSA, Smilek D (2015) Not all mind wandering is created equal: dissociating deliberate from spontaneous mind wandering. Psychol Res 79(5):750–758. 10.1007/s00426-014-0617-x 25284016 10.1007/s00426-014-0617-x

[54] Shaw GA, Giambra L (1993) Task-unrelated thoughts of college students diagnosed as hyperactive in childhood. Dev Neuropsychol 9(1):17–30. 10.1080/87565649309540541

[55] Shrimpton D, McGann D, Riby LM (2017) Daydream believer: Rumination, self-reflection and the temporal focus of mind wandering content. Eur J Psychol 13(4):794–809. 10.5964/ejop.v13i4.1425 29358989 10.5964/ejop.v13i4.1425 PMC5763464

[56] Sibley MH, Pelham WE Jr, Molina BS, Gnagy EM, Waxmonsky JG, Waschbusch DA, Kuriyan AB (2012) When diagnosing ADHD in young adults emphasize informant reports, DSM items, and impairment. J Consult Clin Psychol 80(6):1052–1061 22774792 10.1037/a0029098 PMC3919146

[57] Smallwood J, Schooler JW (2006) The restless mind. Psychol Bull 132(6):946–958. 10.1037/0033-2909.132.6.946 17073528 10.1037/0033-2909.132.6.946

[58] Smith ZR, Breaux RP, Green CD, Langberg JM (2019) Evaluation of the interplay between homework motivation and sluggish cognitive tempo in youth with ADHD: associations with homework performance. J Atten Disord 23(11):1262–1273. 10.1177/1087054718763722 29553294 10.1177/1087054718763722

[59] Thomas R, Sanders S, Doust J, Beller E, Glasziou P (2015) Prevalence of Attention-deficit/hyperactivity disorder: a systematic review and meta-analysis. Pediatrics 135(4):e994–e1001. 10.1542/PEDS.2014-3482 25733754 10.1542/peds.2014-3482

[60] Wammes JD, Seli P, Cheyne JA, Boucher PO, Smilek D (2016) Mind wandering during lectures II: relation to academic performance. Scholarsh Teach Learn Psychol 2(1):33–48. 10.1037/stl0000055

[61] Weiss M, Worling D, Wasdell M (2003) A chart review study of the inattentive and combined types of ADHD. J Atten Disord 7(1):1–9. 10.1177/108705470300700101 14738177 10.1177/108705470300700101

[62] Zadelaar JN, Dekkers TJ, Huizenga HM (2020) The association between risky decision making and attention-deficit/hyperactivity disorder symptoms: a preregistered assessment of need for cognition as underlying mechanism. J Behav Decis Mak 33(5):579–592. 10.1002/bdm.2177

