# Supplementary material for: Does Mind-Wandering Explain ADHD-Related Impairment in Adolescents?
Source: Child Psychiatry Hum Dev. 2023 Jun 29;56(2):346–57. doi: 10.1007/s10578-023-01557-2 (PMC11928403; doi:10.1007/s10578-023-01557-2)
Supplement: Supplementary file 1 — Supplementary file1 (DOCX 45 KB) [file 10578_2023_1557_MOESM1_ESM.docx]

**Supplementary Materials 1**

*This supplementary section describes the results when analyses were performed without outliers, whereas the main manuscript describes analyses including outliers. The overall pattern of results, however, is very similar.*

**Psychometric evaluation** **of the MEWS**

At baseline, the MEWS shows overall high internal consistency for the total sample (Cronbach’s 𝛼 = .92). This did not improve by omitting items. Each item appears to fit well with the total score according to item-total correlations (correlations > .63). Furthermore, the sample shows a high split-half reliability (*r_sb_* = .88). Examining convergent validity at baseline for the total sample, the MEWS correlated moderately to highly with total ADHD scores (*r_s_* = .75, *p* < .001), as well as with inattention (*r_s_* = .67, *p* < .001) and hyperactivity (*r_s_* = .58, *p* < .001). There was an interval of 2-3 months between baseline and follow-up measurement on the MEWS for all participants. Test-retest reliability was moderate/high (*r_s_* = .76, *p* < .001).

**Hierarchical regression analyses**

For all models, *F*-values, *p*-values, *R^2^*, change in *R^2^*, bootstrapped B coefficients, and bootstrapped confidence intervals are reported in Table S1.

***General impairment.*** When mind-wandering was added to the model with only ADHD symptoms, this resulted in a significant increase in explained variance; the model containing ADHD-symptoms and mind-wandering explained 36% of the variance. This was an increase of 6% as compared to the explained variance of the first model containing ADHD-symptoms only. After mind-wandering was added to the model, there still was a smaller but significant association between ADHD symptoms and general impairment, and also a significant association between mind-wandering and general impairment was found.

***Risk-taking behavior.***  We found that the first model (with ADHD symptoms only) was significantly associated with risk-taking behavior, explaining 18% of the variance. Adding mind-wandering to the model increased the explained variance for 1 %. This did not change the association between ADHD symptoms and risk-taking behavior, and there was no association between mind-wandering and risk-taking behavior.

***Homework problems****.* When mind-wandering was added to the first model with only ADHD symptoms, the explained variance did not change significantly. The first model with ADHD symptoms only, was significantly associated with homework problems, explaining 54% of the variance. When mind-wandering was added to the model the explained variance increased to 55%. The relation between ADHD symptoms and homework problems did not change, and there was no association between mind-wandering and homework problems.

***Emotionality****.* Adding mind-wandering to the model with only ADHD symptoms resulted in a significant increase in explained variance; the second model explained 28% of the variance. In the first step of the analyses, we found that ADHD symptoms were significantly associated with emotionality, explaining only 21% of the variance. When mind-wandering was added to the model, the association between ADHD symptoms and emotionality was no longer significant (note that this association was significant in the main manuscript when outliers were not excluded). In this model, we did find a significant association between mind-wandering and emotionality.

***Emotion regulation****.* Adding mind-wandering to the model with only ADHD symptoms, resulted in a significant increase in explained variance; the model explained 14% of the variance. In the first step of the analyses, we found that ADHD symptoms were significantly associated with emotion regulation (i.e., higher levels of ADHD symptoms were related to lower scores on emotion regulation), explaining 11% of the variance. In the second step, with mind-wandering added to the model, we no longer found a significant association between ADHD symptoms and emotion regulation. Instead, we found a significant association between mind-wandering and emotion regulation (i.e., higher levels of mind-wandering were related to poorer emotion regulation).

**Table S1.** Hierarchical regression analyses.

|  |  | Outcome  *Bootstrapped B* | 95% BCI | *F (df)* | *R*^2^ | △*R*^2^ |
| --- | --- | --- | --- | --- | --- | --- |
|  |  | General impairment | |  |  |  |
| Step 1 | ADHD | .55*** | [.49, .60] | 269.08*** (1, 619) | .30 |  |
| Step 2 | ADHD | .25*** | [.16, .35] | 175.41*** (2, 618) | .36 | .06*** |
|  | MEWS | .38*** | [.28, .48] |  |  |  |
|  |  | Risk-taking behaviors | |  |  |  |
| Step 1 | ADHD | .39*** | [.30, .49] | 65.83*** (1,302) | .18 |  |
| Step 2 | ADHD | .49*** | [.35, .63] | 34.84*** (2,301) | .19 | .01 |
|  | MEWS | -.13 | [-.26, .01] |  |  |  |
|  |  | Homework problems | |  |  |  |
| Step 1 | ADHD | .65*** | [.58, .72] | 371.03*** (1,296) | .56 |  |
| Step 2 | ADHD | .67*** | [.57, .78] | 185.34*** (2,295) | .56 | .001 |
|  | MEWS | -.03 | [-.14, .06] |  |  |  |
|  |  | Emotionality |  |  |  |  |
| Step 1 | ADHD | .46*** | [.35, .58] | 80.62*** (1, 307) | .21 |  |
| Step 2 | ADHD | .14 | [-.01, .30] | 60.29*** (2, 306) | .28 | .08*** |
|  | MEWS | .42*** | [.28, .55] |  |  |  |
|  |  | Emotion regulation | |  |  |  |
| Step 1 | ADHD | -.29*** | [-.38, -.19] | 36.67*** (1,296) | .11 |  |
| Step 2 | ADHD | -.12 | [-.26, .02] | 24.16*** (2, 295) | .14 | .03** |
|  | MEWS | -.23** | [-.38, -.07] |  |  |  |

*Note*. All variables reflect sum scores. The table shows standardized bootstrapped regression coefficients where general impairment, risk-taking behaviors, homework problems, emotionality, and emotion regulation are regressed on the ADHD symptom score and the MEWS score. Step 1 of each regression model shows association of ADHD symptom score with all outcome measures. Step 2 includes two predictors, ADHD and MEWS, and shows the additional contribution of mind-wandering in association with all five outcome measures. BCI = Bootstrapped Confidence Interval, Df = degrees of freedom, MEWS = Mind Excessively Wandering Scale. * *p* < .05, ** *p* < .01, *** *p* < .001

**Table S2.** Hierarchical regression analyses (inattention instead of ADHD).

|  |  | Outcome  *Bootstrapped B* | 95% BCI | *F (df)* | *R*^2^ | △*R*^2^ |
| --- | --- | --- | --- | --- | --- | --- |
|  |  | General impairment | |  |  |  |
| Step 1 | Inattention | .53*** | [.46, .58] | 243.13*** (1, 619) | .28 |  |
| Step 2 | Inattention | .22*** | [.12, .32] | 172.52*** (2, 618) | .36 | .08*** |
|  | MEWS | .41*** | [.31, .51] |  |  |  |
|  |  | Risk-taking behaviors | |  |  |  |
| Step 1 | Inattention | .33*** | [.24, .42] | 49.85*** (1,302) | .14 |  |
| Step 2 | Inattention | .37*** | [.23, .52] | 25.17*** (2,301) | .14 | .00 |
|  | MEWS | -.06 | [-.20, .10] |  |  |  |
|  |  | Homework problems | |  |  |  |
| Step 1 | Inattention | .71*** | [.65, .77] | 455.65*** (1,296) | .61 |  |
| Step 2 | Inattention | .71*** | [.62, .80] | 227.08*** (2,295) | .61 | .00 |
|  | MEWS | -.01 | [-.10, .08] |  |  |  |
|  |  | Emotionality | |  |  |  |
| Step 1 | Inattention | .44*** | [.33, .55] | 63.69*** (1, 303) | .17 |  |
| Step 2 | Inattention | .07 | [-.08, .22] | 59.20*** (2, 302) | .28 | .11*** |
|  | MEWS | .51*** | [.37, .63] |  |  |  |
|  |  | Emotion regulation | |  |  |  |
| Step 1 | Inattention | -.26*** | [-.35, -.17] | 32.49*** (1, 296) | .10 |  |
| Step 2 | Inattention | -.08 | [-.22, .05] | 23.49*** (2, 295) | .14 | .04*** |
|  | MEWS | -.25** | [-.40, -.10] |  |  |  |

*Note*. All variables reflect sum scores. The table shows standardized bootstrapped regression coefficients where general impairment, risk-taking behaviors, homework problems, emotionality, and emotion regulation are regressed on the inattention symptom score and the MEWS score. Step 1 of each regression model shows the association of the inattention symptom score with all outcome measures. Step 2 includes two predictors, inattention and MEWS, and shows the additional contribution of mind-wandering in association with all five outcome measures. BCI = Bootstrapped Confidence Interval, Df = degrees of freedom, MEWS = Mind Excessively Wandering Scale. * *p* < .05, ** *p* < .01, *** *p* < .001.

**Supplementary Materials 2**

Below are the Dutch items of the MEWS, which were translated from Mowlem et al. (2016). The original MEWS consisted of 15 items, based on their psychometric study Mowlem and colleagues recommended to exclude three items when using the MEWS in future research. Therefore, our Dutch version has 12 items.

1. Ik heb moeite mijn gedachten onder controle te houden
2. Ik vind het moeilijk mijn gedachten uit te zetten
3. Ik heb twee of meer verschillende gedachten op hetzelfde moment
4. Mijn gedachten zijn ongeorganiseerd en gaan alle kanten op
5. Mijn gedachten gaan de hele tijd door
6. Ik ervaar onophoudelijk mentale activiteit
7. Ik vind het moeilijk om aan één ding te denken zonder dat er een andere gedachte in mijn hoofd opkomt
8. Ik vind mijn gedachten afleidend en ze voorkomen dat ik kan focussen op wat ik aan het doen ben
9. Ik heb moeite mijn gedachten af te remmen en mij op één ding tegelijk te focussen
10. Ik vind het moeilijk om helder na te denken, alsof mijn hoofd wazig is
11. Ik merk dat ik heen en weer fladder tussen verschillende gedachten
12. Ik kan mijn gedachten alleen op één ding tegelijk focussen met aanzienlijke moeite

**Supplementary Materials 3**

**Table S3 Item-total correlations MEWS**

| Item | Corrected item-total correlation |
| --- | --- |
| MEWS 1. | .692 |
| MEWS 2. | .699 |
| MEWS 3. | .649 |
| MEWS 4. | .750 |
| MEWS 5. | .626 |
| MEWS 6. | .693 |
| MEWS 7. | .736 |
| MEWS 8. | .723 |
| MEWS 9. | .757 |
| MEWS 10. | .660 |
| MEWS 11. | .742 |
| MEWS 12. | .714 |

*Note*. The item-total correlation measures the correlation between each MEWS item and the MEWS total score, with the item in question excluded.

**Supplementary Materials 4**

**Table S4.1.** Hierarchical regression analyses for boys only

|  |  | Outcome  *Bootstrapped B* | 95% BCI | *F (df)* | *R*^2^ | △*R*^2^ |
| --- | --- | --- | --- | --- | --- | --- |
|  |  | General impairment (*n*=222) | |  |  |  |
| Step 1 | ADHD | .65*** | [.55, .75] | 152.20*** (1, 220) | .41 |  |
| Step 2 | ADHD | .50*** | [.33, .67] | 80.15*** (2, 219) | .42 | .01* |
|  | MEWS | .19* | [.02, .36] |  |  |  |
|  |  | Risk-taking behaviors (*n*=105) | |  |  |  |
| Step 1 | ADHD | .59*** | [.33, .85] | 29.39*** (1,103) | .22 |  |
| Step 2 | ADHD | .55* | [.05, 1.02] | 14.61*** (2,102) | .22 | .00 |
|  | MEWS | .05 | [-.36, .50] |  |  |  |
|  |  | Homework problems (*n*=119) | |  |  |  |
| Step 1 | ADHD | .73*** | [.61, .86] | 146.58*** (1,117) | .56 |  |
| Step 2 | ADHD | .82*** | [.64, .99] | 74.51*** (2,116) | .56 | .01 |
|  | MEWS | -.12 | [-.28, .07] |  |  |  |
|  |  | Emotionality (*n*=105) | |  |  |  |
| Step 1 | ADHD | .49*** | [.27, .67] | 26.37*** (1, 103) | .20 |  |
| Step 2 | ADHD | .09 | [-.21, .38] | 20.92*** (2, 102) | .29 | .09*** |
|  | MEWS | .50*** | [.25, .76] |  |  |  |
|  |  | Emotion regulation (*n*=105) | |  |  |  |
| Step 1 | ADHD | -.26* | [-.46, -.05] | 5.51* (1,103) | .05 |  |
| Step 2 | ADHD | -.00 | [-.36, .35] | 4.58* (2, 102) | .08 | .03 |
|  | MEWS | -.32 | [-.68, .02] |  |  |  |

*Note*. All variables reflect sum scores. The table shows standardized bootstrapped regression coefficients where general impairment, risk-taking behaviors, homework problems, emotionality, and emotion regulation are regressed on the ADHD symptom score and the MEWS score. Step 1 of each regression model shows association of ADHD symptom score with all outcome measures. Step 2 includes two predictors, ADHD and MEWS, and shows the additional contribution of mind-wandering in association with all five outcome measures. BCI = Bootstrapped Confidence Interval, Df = degrees of freedom, MEWS = Mind Excessively Wandering Scale. * *p* < .05, ** *p* < .01, *** *p* < .001.

**Table S4.2.** Hierarchical regression analyses for girls only

|  |  | Outcome  *Bootstrapped B* | 95% BCI | *F (df)* | *R*^2^ | △*R*^2^ |
| --- | --- | --- | --- | --- | --- | --- |
|  |  | General impairment (*n*=396) | |  |  |  |
| Step 1 | ADHD | .52*** | [.45, .59] | 158.79*** (1,394) | .29 |  |
| Step 2 | ADHD | .20** | [.07, .32] | 110.59*** (2,393) | .36 | .07*** |
|  | MEWS | .43*** | [.29, .56] |  |  |  |
|  |  | Risk-taking behaviors (*n*=203) | |  |  |  |
| Step 1 | ADHD | .43*** | [.32, .55] | 61.69*** (1,201) | .24 |  |
| Step 2 | ADHD | .50*** | [.34, .66] | 31.54*** (2,200) | .24 | .01 |
|  | MEWS | -.09 | [-.25, .06] |  |  |  |
|  |  | Homework problems (*n*=193) | |  |  |  |
| Step 1 | ADHD | .78*** | [.69, .87] | 389.36*** (1,191) | .67 |  |
| Step 2 | ADHD | .72*** | [.57, .88] | 195.44*** (2,190) | .67 | .00 |
|  | MEWS | .08 | [-.10, .24] |  |  |  |
|  |  | Emotionality (*n*=203) | |  |  |  |
| Step 1 | ADHD | .45*** | [.30, .61] | 59.81*** (1, 201) | .23 |  |
| Step 2 | ADHD | .19* | [.02, .38] | 40.63*** (2, 200) | .29 | .06*** |
|  | MEWS | .34*** | [.20, .49] |  |  |  |
|  |  | Emotion regulation (*n*=203) | |  |  |  |
| Step 1 | ADHD | -.22* | [-.37, -.06] | 10.26** (1,201) | .05 |  |
| Step 2 | ADHD | -.02 | [-.23, .18] | 8.78*** (2, 200) | .08 | .03** |
|  | MEWS | -.26** | [-.46, -.07] |  |  |  |

*Note*. All variables reflect sum scores. The table shows standardized bootstrapped regression coefficients where general impairment, risk-taking behaviors, homework problems, emotionality, and emotion regulation are regressed on the ADHD symptom score and the MEWS score. Step 1 of each regression model shows association of ADHD symptom score with all outcome measures. Step 2 includes two predictors, ADHD and MEWS, and shows the additional contribution of mind-wandering in association with all five outcome measures. BCI = Bootstrapped Confidence Interval, Df = degrees of freedom, MEWS = Mind Excessively Wandering Scale. * *p* < .05, ** *p* < .01, *** *p* < .001.
